# The mitochondrial genome and phylogenetic analysis of *Rhacophorus rhodopus*

**DOI:** 10.1038/s41598-022-17814-8

**Published:** 2022-08-11

**Authors:** Wei Chen, Haifen Qin, Zhenkun Zhao, Jiahong Liao, Hongzhou Chen, Lichun Jiang, Buddhi Dayananda

**Affiliations:** 1 School of Resources and Environmental Engineering, Anhui University, Hefei, 230601 China; 2 Key Laboratory for Molecular Biology and Biopharmaceutics, School of Life Science and Technology, Mianyang Normal University, Mianyang, 621000 Sichuan China; 3 School of Agriculture and Food Sciences, The University of Queensland, Brisbane, QLD 4072 Australia

**Keywords:** DNA sequencing, Molecular evolution

## Abstract

Classification of the genus *Rhacophorus* has been problematic. In particular there has been considerable controversy surrounding the phylogenetic relationships among *Rhacophorus rhodopus*, *R. bipunctatus,* and *R. reinwardtii*. To examine the relationship among these *Rhacophorus* species, we assembled the complete mitochondrial genome sequence of *R. rhodopus*. The *R. rhodopus* genome is 15,789 bp in length with 12 protein-coding genes (PCGs) (losing ND5), two ribosomal genes, 22 transfer RNA genes, and a control region (D-loop). Base composition of the overall sequence was 60.86% for A + T content and 39.14% for C + G content. Most of the PCGs used ATG as a start codon, except for the COX I gene, which used the ATA start codon. COX I and ND6 used AGG and ATP8 stop codons respectively, while ND3 and ND4L used the TAA stop codon. For the remaining seven genes, the stop codons was incomplete. In addition, both 5' and 3' of the control areas had distinct repeating regions. Based on three datasets and two methods (Bayesian inference (BI) and maximum likelihood (ML)), we reconstructed three phylogenetic trees to explore the taxonomic status of the species and the phylogenetic relationship among *R. rhodopus*, *R. bipunctatus* and *R. reinwardtii*. Our results indicated that these three species are non-monophyletic; thus, the phylogenetic relationship among them is complex and difficult to determine. Further, *R. rhodopus* is divided into three lineages from different parts of China. The two *Rhacophorus* samples showed very close phylogenetic relationship with *R. rhodopus*. Our results add to the mitochondrial genome database of amphibians and will help to disentangle the phylogenetic relationships within the Rhacophoridae.

## Introduction

Mitochondria are important functional organelles within eukaryotic cells and mitochondrial DNA (mtDNA) is the small circular chromosome found inside mitochondria^1^. As an important molecular marker, mtDNA exhibits valuable characteristics including high mutation and substitution rates, rare gene recombination, maternal transmission pathway, high copy number, and easy accessibility^2^. Hence, it has been widely used in phylogenetic analyses and phylogeographic studies^3,4^. Furthermore, mtDNA has been used to test microevolutionary processes and to investigate population genetic structure and identification of cryptic species^5^.

Typically, vertebrate mtDNA tends to be conserved, with 37 genes including 13 protein-coding genes, two ribosomal RNAs (rRNA), 22 transfer RNAs (tRNA), and a control region^6^, with a size range from 15 to 21 kb^7^. However, gene rearrangements^8^ have often been reported in anuran mitogenomes due to gene losses^9^, gene transpositions^10^, and gene duplication^11,12^. To detect whether these gene rearrangements are universal in anurans, comparative studies using mtDNA from a wide variety of anuran species are needed.

The family Rhacophoridae is one of the most abundant and ecologically diverse group of anurans worldwide^13^, and contains 443 recognized species of 23 genera^14^. In China, 94 species from 14 known genera are recorded (AmphibiaChina, 2021). Within the Rhacophoridae, *Rhacophorus rhodopus* is widely distributed in southeast Asia including China (southeastern Xizang, southern Yunnan, northeastern Guangxi, and Hainan)^14,15^. In the past few decades the phylogenetic relationship between *R. rhodopus* and *R. bipunctatus* has attracted considerable controversy*.* For example*,* Inger et al. and Frost suggest that *R. rhodopus* is a synonym of *R. bipunctatus*^16,17^, but subsequently Bordoloi et al. consider *R. rhodopus* as a valid species^18^. Moreover, the phylogenetic relationships among *R. bipunctatus*, *R. rhodopus* and *R. reinwardtii* have also been disputed*.* Wilkinson et al. suggest that *R. bipunctatus* is more closely related to *R. reinwardtii* than to *R. rhodopus*^19^. Conversely, Yu et al. argue that *R. bipunctatus* is more closely related to *R. rhodopus* than to *R. reinwardtii*^20,21^. Currently, the phylogenetic status of *R. rhodopus* and the phylogenetic relationship among *R. rhodopus, R. bipunctatus* and *R. reinwardtii* remains unclear.

**Figure 1:**
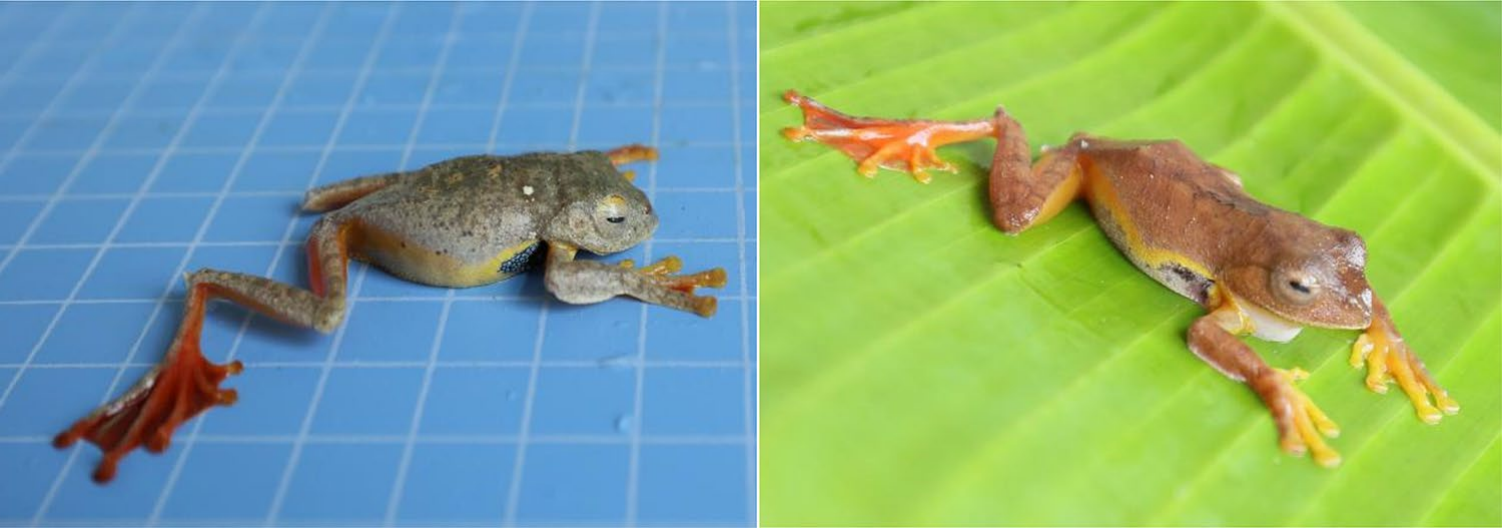
Rhacophorus species collected from Motuo County in China.

Further controversy surrounds *Rhacophorus* species from the Tibetan region of China. Chen et al. suggested that *Rhacophorus* species from the Motuo Tibet region are *R. bipunctatus* due to their morphological similarities^13^, but other researchers believe they are *R. rhodopus*^22^. During our field investigations, we found two *Rhacophorus* species with different morphological characters (Fig. 1). One specimen was morphologically similar to *R. bipunctatus* and the other was morphologically similar to *R. rhodopus*. Since amphibian morphology and skin colour often vary according to the external environment, it is difficult to identify the species based on morphological characteristics alone. Fortunately, molecular evidence, such as complete mitochondrial genome (mtDNA) sequences, can be used to delineate species and solve conflicting evolutionary histories^23^.

We aimed to 1) identify *Rhacophorus* species from Motuo County in the Tibet Autonomous Region, China, based on mtDNA, 2) provide references for future genome research and 3) determine the phylogenetic relationships within the Rhacophoridae and also the phylogenetic status of *R. rhodopus, R. bipunctatus* and *R. reinwardtii*. In order to achieve those aims, we focused on more extensive classification samplings within the Rhacophoridae. Additionally, we included GenBank sequences to explore detailed mitogenome characteristics and phylogenetic relationships within the Rhacophoridae.

## Results and discussion

Based on mitogenome evidence, we found that the two Rhacophorus individuals we collected in Motuo County in the Tibet Autonomous Region, China were *R. rhodopus* (Figs. 5, 6 and 7)*.* The two sequences have same gene order and both of them lose ND5gene, but one sequences (OK165559) is shorter than our reported sequences (OK181853). In our reported sequence, there are two more bases in the 16rRNA, and 37 more bases in D-loop. Here we only analyzed the structural characteristics of one sequences (OK181853).

### Structural characteristics of the mitochondrial genome

**Figure 2:**
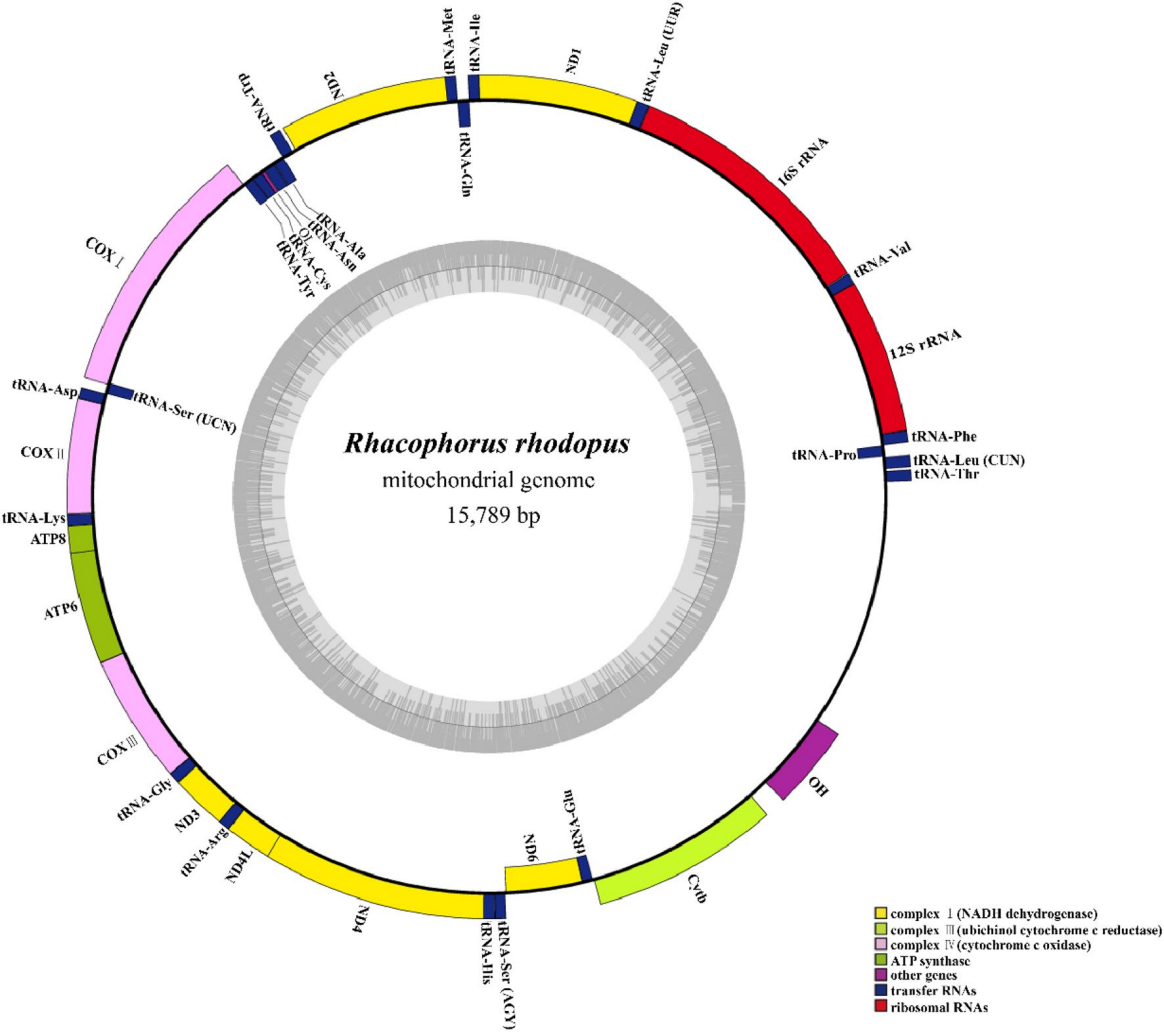
The complete mitochondrial genome sequence of *Rhacophorus rhodopus* collected from Motuo County in China.

We determined that the complete mitochondrial genome of *R. rhodopus* is 15,789 bp in length, and consists of 12 protein-coding genes (PCGs) (losing NADH dehydrogenase subunit 5), two ribosomal RNA genes (rRNAs), 22 transfer RNA genes (tRNAs), and a control region (D-loop) (Fig. 2 and Table S1). Among the 36 fragment genes, ND6 and eight genes (tRNA-*Pro*, tRNA-*Gln*, tRNA-*Ala*, tRNA-*Asn*, tRNA-*Cys*, tRNA-*Tyr*, tRNA-*Ser* (UCN) and tRNA-*Glu*) were on the light strands (L-strand), and the remainder were located on the heavy strands (H-strand) (Fig. 2 and Table S1).

The mitogenome structure of *R. rhodopus* was conserved, similar to the gene sequence structures of *Rhacophorus schlegelii*^24^ and *Rhacophorus dennysi*^4^. Four tRNA genes (tRNA-*Thr*, tRNA-*Leu*(CUN), tRNA-*Pro* and tRNA-*Phe*) formed a TLPF tRNA cluster, different from the neobatrachian-type arrangement^25^. In Rhacophoridae species, the ND5 gene is located between D-loop and tRNA-*Thr*^4^, but in *R. rhodopus* mtDNA, we found that the ND5 gene was lost. We also used the ND5 of other frog species to blast with *R. rhodopus* mtDNA and yet we did not find the ND5.The ND5 gene loss was also observed in *Polypedates megacephalus*^9^. This phenomenon may be common in vertebrates^9,25^.

The complete genome of *R. rhodopus* mtDNA consisted of 30.83% A, 30.03% T, 14.81% G, and 24.32% C. Similar to the base distribution in other anurans^21^, the A + T content (60.86%) was higher than the G + C content (39.14%), showing an obvious preference for A + T in the complete mitogenome sequences of this species. We also found that the AT-skew was 0.013 and the GC-skew was − 0.243, indicating more A than T, and more C than G (Table S2).

### Protein-coding genes and codon usage patterns

Within the complete mitogenome genome of *R. rhodopus*, the total length of the 12 PCGs was 9,519 bp. Among the 12 PCGs, 11 PCGs (except COX I) used ATG as the initiation codon, while COX I genes initiated with an ATA codon. For COX I and ND6 the stop codon was AGG, whereas for ND3 and ND4L the stop codon was TAA. Seven protein genes (ATP6, COX II, COXIII, ND1, ND2, ND4 and Cytb) ended with incomplete stop codons TA- or T-- (Table S2). These T--/TA- stop codons become a complete TAA stop codon through the post-transcriptional polyadenylation^26^.

The AT/CG-skews of the 12 PCGs are listed in Table S3. Except for ND2, ND6 and COX II, the remaining nine PCGs were negative in both the AT-skew and GC-skew. Meanwhile, the A + T content of the 12 PCGs was 60.37%; the AT-skew (− 0.074) and the GC-skew (− 0.253) were negative. The codon usage and relative synonymous codon usage (RSCU) values of *R. rhodopus* are shown in Table S3. Eight of 64 codons showed the highest use frequency and they were AUU (192), UUU (161), CUA (141), UUA (133), AUA (133), ACA (105), GCC (100), CUU (100). However, UCG and CGG codons were the least used stop codon.

### Ribosomal RNA and transfer RNA genes

Similar to other vertebrates^21^, the mitogenome of *R. rhodopus* also included 12S and 16S rRNA genes, which were on the H strand. The 12S rRNA gene was 935 bp long and located between tRNA-*Phe* and tRNA-*Val* genes. The 16S rRNA gene with a length of 1,572 bp was located between tRNA-*Val* and tRNA-*Leu* (UUR) genes (Table S1). We found that the content of A + T (12S rRNA genes 54.55%, 16S rRNA genes 60.56%) was higher than that of C + G (12S rRNA genes 45.45%, 16S rRNA genes 39.44%). The AT-skew was slightly positive whereas the GC-skew was strongly negative (Table S2).

**Figure 3:**
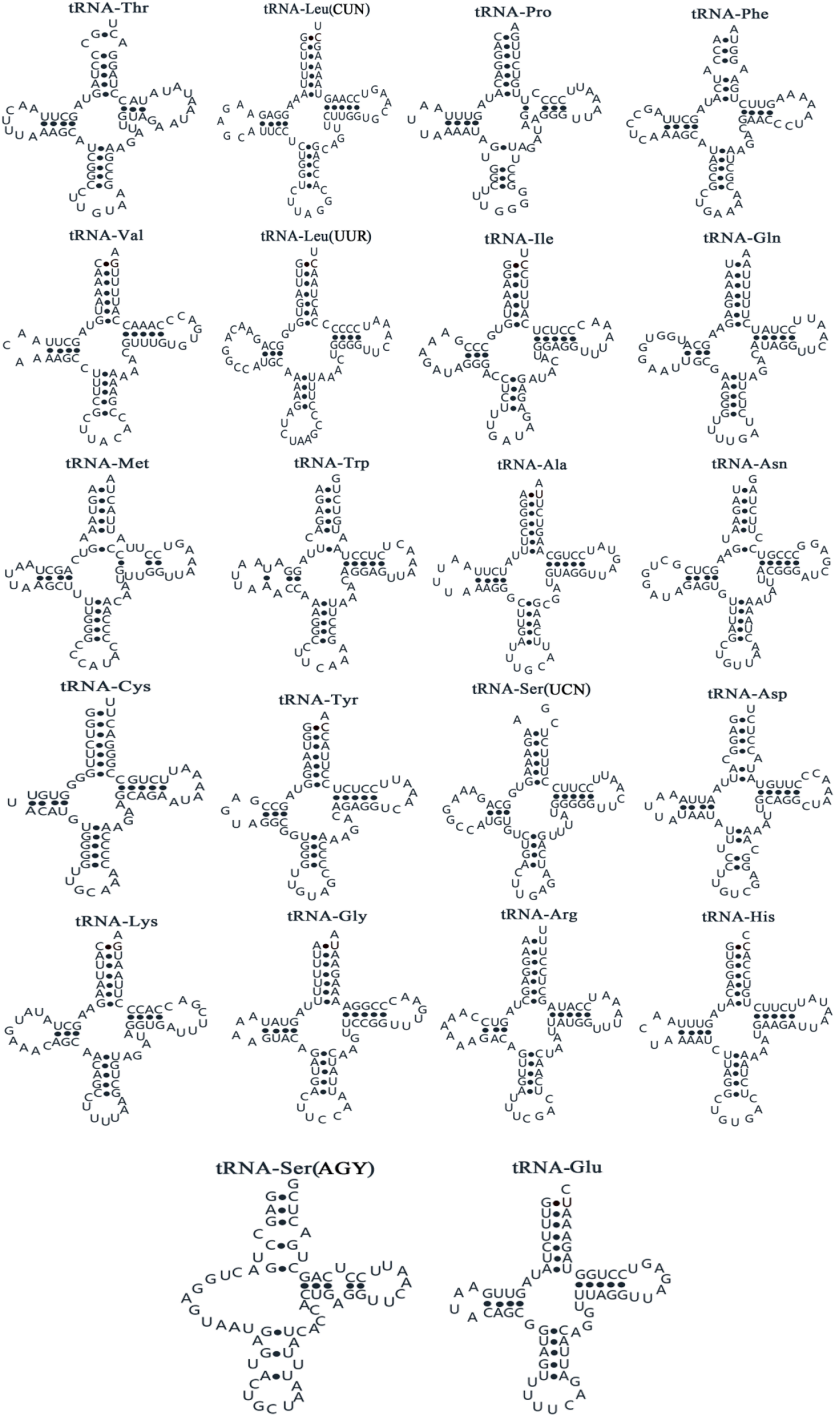
Putative tRNA secondary structures found mitochondrial genome of *Rhacophorus rhodopus.*

Of the 22 tRNA genes identified in the *R. rhodopus* mitogenome, 14 genes were located on the H strand and 8 genes were located on the L strand (Table S1). The secondary structure of tRNA is shown in Fig. 3. We found that 21 tRNA genes, except for tRNA-*Ser* (AGY), were able to form the classical cloverleaf secondary structure and that the use of anticodon was the same in other vertebrates^3^. In contrast, the tRNA-*Ser* (AGY) gene was unable to form a cloverleaf structure due to a lack of a dihydrouridine (DHU) arm. This is a common phenomenon in vertebrates^27^. However, Cheng et al.suggested that lack of DHU could become functional by adjusting its structural conformation to fit the ribosome in a similar way to that of usual tRNAs in the ribosomes^28^.

### Noncoding regions

We identified two major noncoding regions in the *R. rhodopus* mitochondrial genome, at the origin of L-strand replication (OL) and in the control region (D-loop). The OL, a length of 23 bp, was located between tRNA-*Asn* and tRNA-*Cys* in the WANCY genes cluster (Table S1). The D-loop (2,230 bp), which was the longest part in the complete genome, was located between the Cytb gene and the tRNA-*Thr* gene on the H-strand (Table S1). Keddie et al. speculated that the D-loop may play an important role in gene replication^29^. In the D-loop sequence, A + T content was 67.17% and G + C content was 32.83%. In addition, AT/CG-skew analysis showed that the D-loop gene of *R. rhodopus* has a positive AT-skew (0.021), while the GC-skew (-0.180) was strongly negative (Table S2).

**Figure 4:**
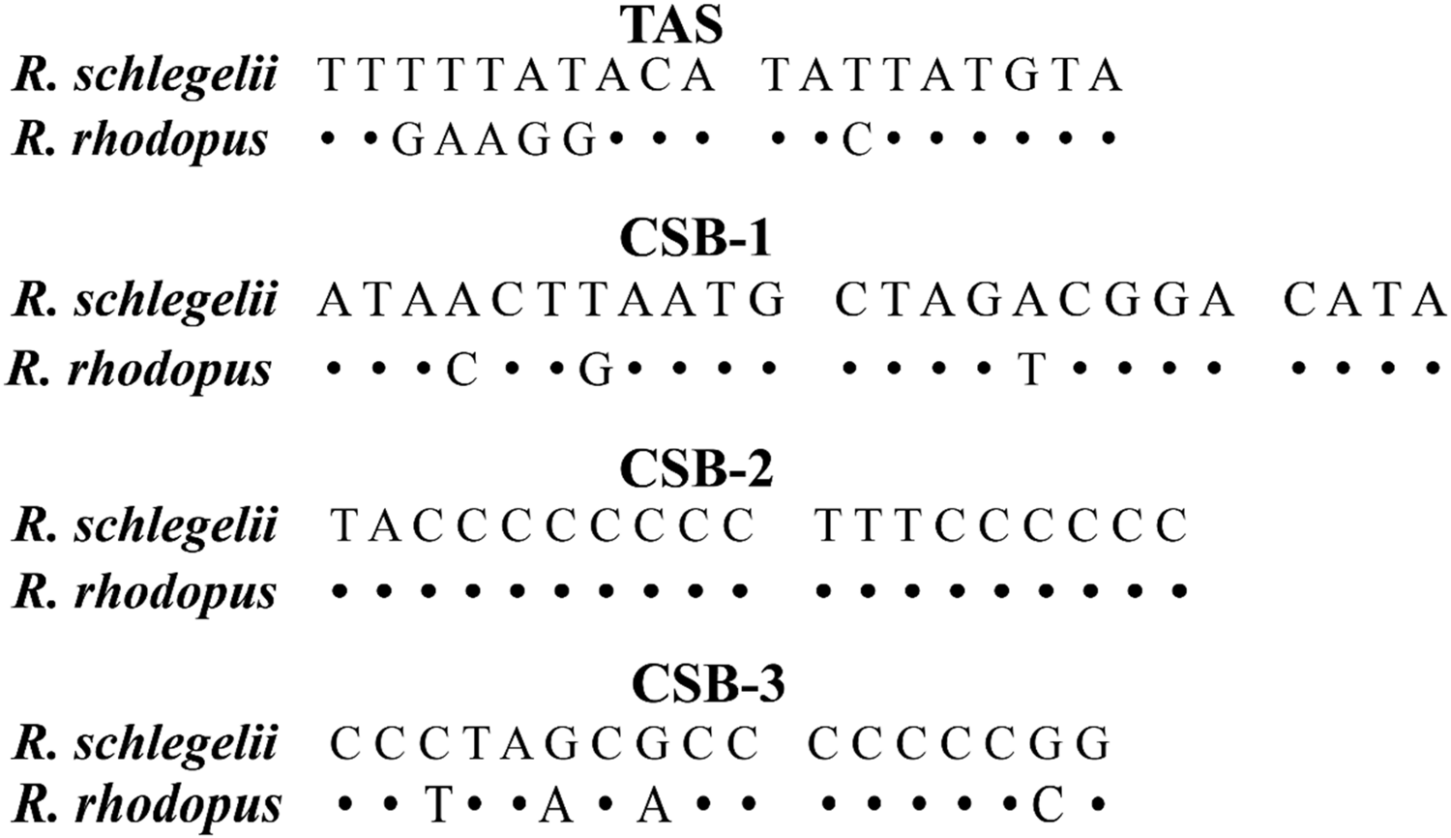
Structures and alignments of identified putative termination-associated sequences (TAS) and conserved sequence blocks (CSB1-3). Alignment gaps and nucleotides identical to the first line are indicated by a dot (**·**), respectively. Variable nucleotides are marked with corresponding nucleotides.

In general, the control regions contained several specific components, which can be easily identified by two tandem repeat units at two ends. We found that both 5′ and 3′-sides of the D-loop had two obvious repeat regions. One was 38 bp of 13.8 tandem repeat units (5′-TTGAAGGACA TACTATGTAT AATCACCATA TACTATGC-3′) on the 5′-side end, and the other was a 11 bp of 12.1 tandem repeat units (5′-TATATATGTAA-3′) on the 3′-side. In addition, three conserved sequence blocks (CSBs) were also detected (24 bp CSB-1, 5′-ATACCTGAAT GCTAGACGGA CATA-3′; 19 bp CSB-2, 5′-TACCCCCCCC TTTCCCCCC-3′; 17 bp CSB-3, 5′-CCTTAACACC CCCCCCG-3′) (Fig. 4 and Table S4). This phenomenon had also been observed in D-loops of other anuran species^3,9^.

### Phylogenetic relationships

**Figure 5:**
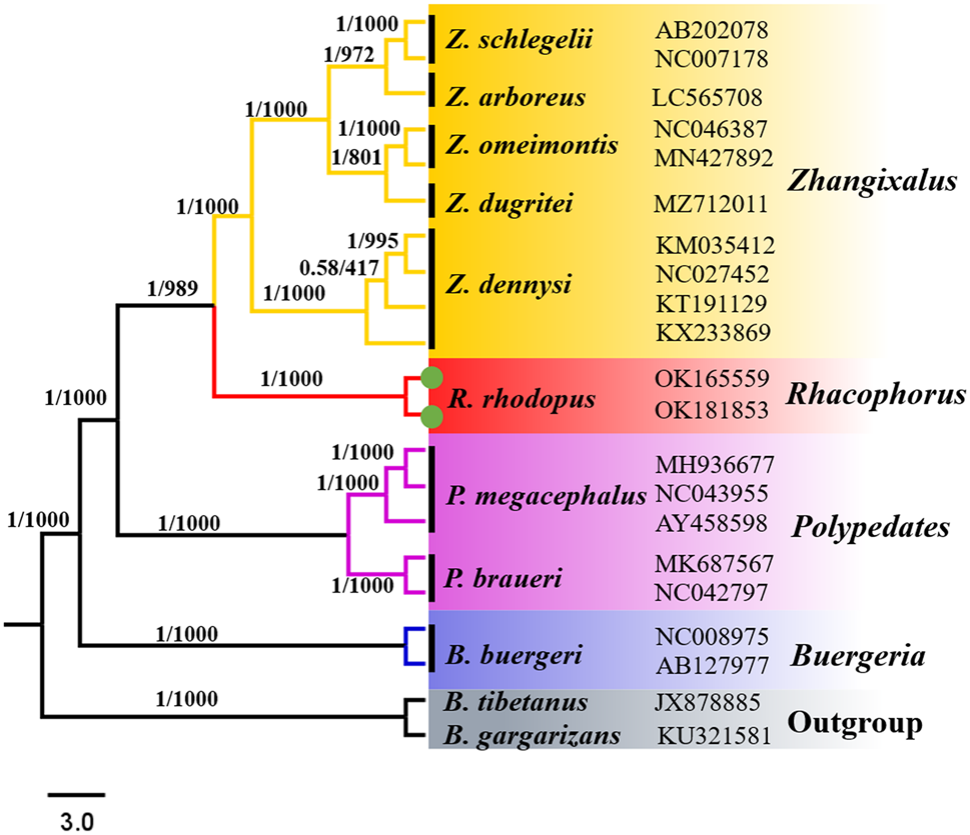
BI and ML analysis of 11 species complete mitochondrial genome sequence, *Bufo gargarizans* and *Bufo tibetanus* as outgroups. Tree topologies produced by BI and ML analyses were equivalent. Bayesian posterior probability (PP) and bootstrap support (BP) values for ML analyses are shown in order on the nodes.

**Figure 6:**
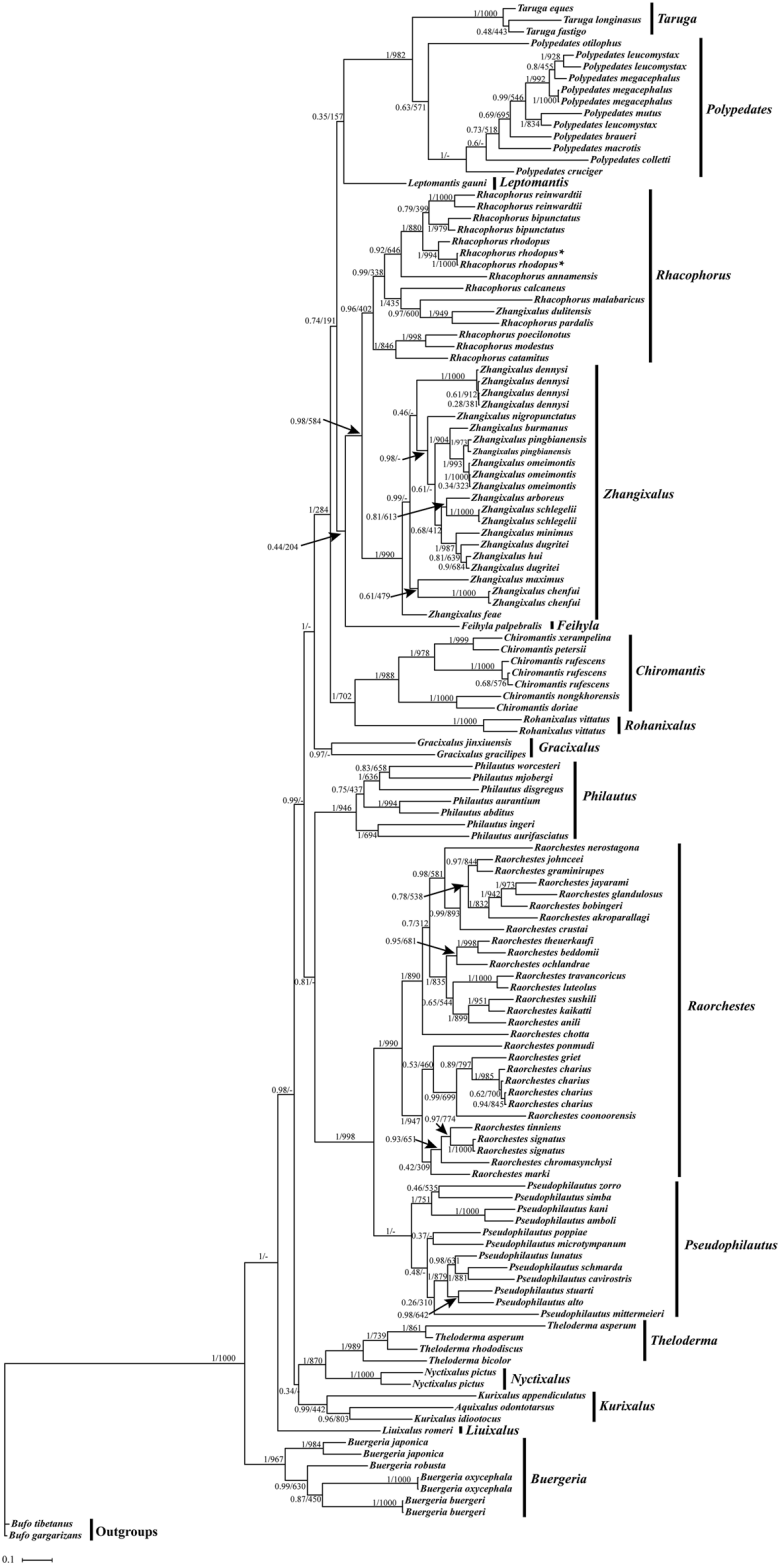
BI and ML analysis of 102 species based on 12S + 16S + CYTB genes sequence. *Bufo gargarizans* and *Bufo tibetanus* as outgroups. Tree topologies produced by BI and ML analyses were equivalent. Bayesian posterior probability (PP) and bootstrap support (BP) values for ML analyses are shown in order on the nodes.

**Figure 7:**
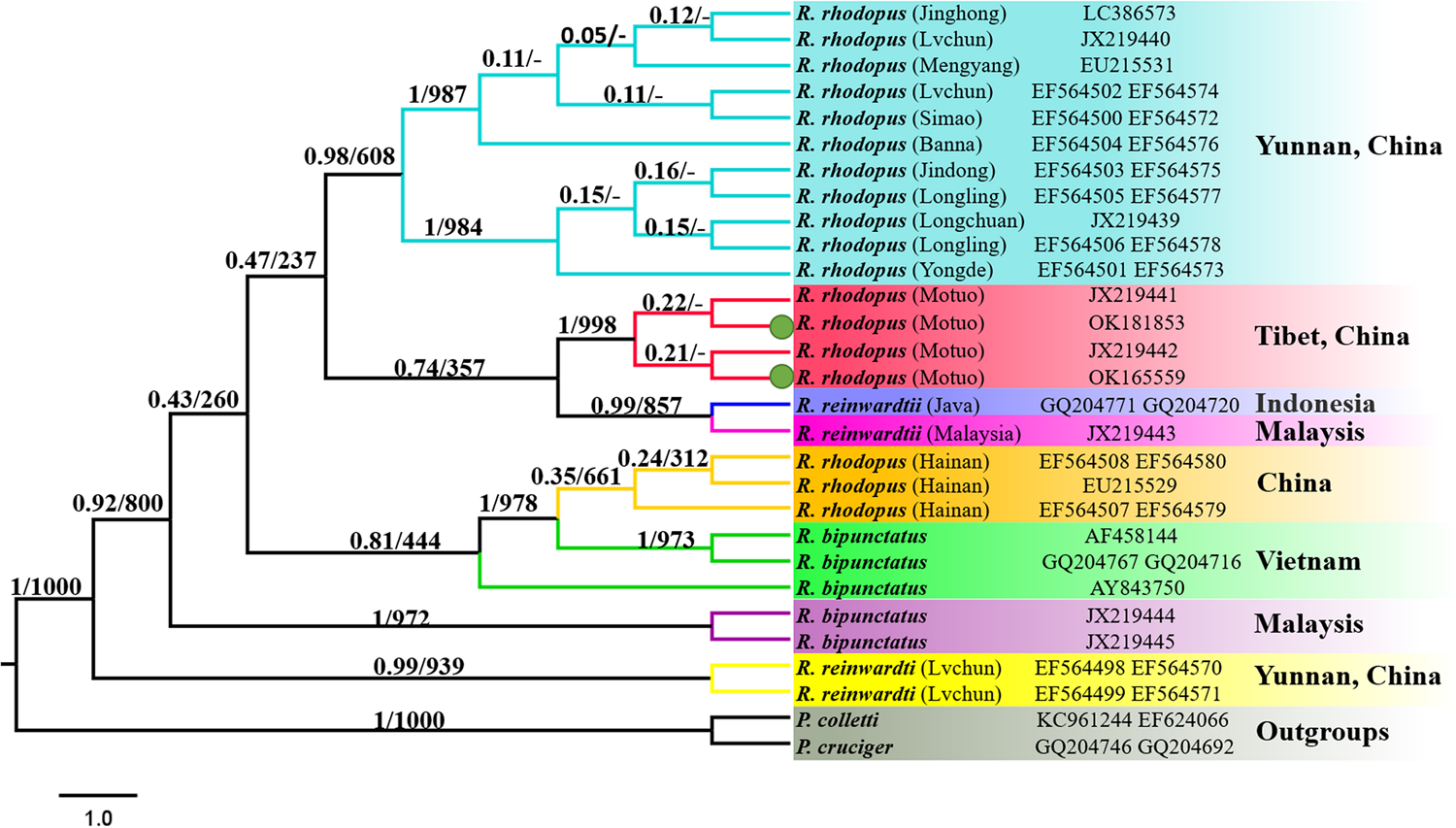
BI and ML analysis of *Rhacophorus rhodopus*, *Rhacophorus bipunctatus* and *Rhacophorus reinwardtii,* based on 12S and 16S. *Polypedates colletti* and *Polypedates cruciger* as outgroups. Tree topologies produced by BI and ML analyses were equivalent. Bayesian posterior probability (PP) and bootstrap support (BP) values for ML analyses are shown in order on the nodes.

We reconstructed BI and ML phylogenetic trees with three types of datasets, and both BI and ML phylogenetic trees showed similar topologies. Hence, we only show the BI tree in this study (Figs. 5, 6 and 7).

### Phylogenetic analysis based on the long mitochondrial genome data set

Our results show that *Zhangixalus, Rhacophorus* and *Polypedates* form a monophyletic group (PP = 1.00, BP = 1000), which supports the monophyletic origin of the tree frog family^44^. The phylogenetic tree is divided into two main branches. One separate branch is the genus *Buergeria*, the other main branch contains *Zhangixalus, Rhacophorus* and *Polypedates* (Fig. 5). Our results show that (*Zhangixalus* + *Rhacophorus*) is a sister clade of *Polypedates* (PP = 1.00, BP = 1000), which is consistent with a previous Rhacophoridae phylogenetic analyses^4^. In the *Rhacophorus* clade, two *R. rhodopus* from Tibet formed a clade with strong supports (PP = 1.00, BP = 1000) and the clade of *R. rhodopus* appeared as the sister taxon to the *Z. dennysi* + ((*Z. omeimontis* + *Z. dugritei*) + (*Z. schlegelii* + *Z. arboreus*)) clade (PP = 1.00, BP = 989). Here, *Z. dennysi* forms a monophyletic group, with strong support values (PP = 1.00, BP = 1000), which are similar to the results based on 16S rRNA gene phylogenetic analyses^30^.

### *Phylogenetic analysis based on the concatenated 12S* + *16S* + *Cytb genes data set*

We reconstructed BI and ML phylogenetic trees according to the concatenated 12S + 16S + Cytb gene dataset from 396 sequences of 104 Rhacophoridae species retrieved from NCBI (Fig. 6). Our results revealed monophyly of seven genera (*Polypedates*, *Taruga*, *Kurixalus*, *Pseudophilautus*, *Theloderma*, *Raorchestes,* and *Buergeria*) in the Rhacophoridae^30^. Previously, the genus *Rhacophorus* was divided into the genus *Rhacophorus*, *Leptomantis* and *Zhangixalus*^31^. However, in our study only *Rhacophorus* and *Zhangixalus* formed a clade*.* Furthermore, we found that *R. pardalis* and *Z. dulitensis* formed a sister group which was similar to the privious phylogenetic analysis of Meegaskumbura et al. and Chan et al.^32,33^. Our results also showed that *R. bipunctatus* and *R. reinwardtii* are more closely related than *R. rhodopus*. Thus, this result is consistent with Wilkinson et al.^19^, but does not support that *R. bipunctatus* was more closely related to *R. rhodopus*^20,21^.

### Phylogenetic analysis of different populations of R. rhodopus based on the concatenated 12S and 16S rRNA genes dataset

To further explore the phylogenetic relationship of *R. rhodopus* in Tibet, we reconstructed BI and ML phylogenetic trees according to the concatenated 12S + 16S genes (842 bp) of *R. rhodopus*, *R. reinwardtii* and *R. bipunctatus* from different populations. The BI tree revealed that the three Rhacophorus individuals from the Motuo Tibet populations form a monophyletic clade, suggesting that they are the same species (*R. rhodopus*), which is consistent with the findings of Li et al.^22^. In addition, our results show that the two *R. reinwardtii* individuals from Yunnan China forms an independent branch, whereas *R. reinwardtii* from Indonesia and Malaysia, and the three *R. rhodopus* individuals from the Motuo Tibet populations also form a sister clade (Fig. 6). This suggests that *R. reinwardtii* may be paraphyletic^34^. Hence, the relationship among *R. rhodopus*, *R. bipunctatus*, and *R. reinwardtii* cannot be reliably solved and further research will explore the morphological features of *R. reinwardtii* and to reconfirm the accuracy of sequences of *R. reinwardtii* from Indonesia, Malaysia and from Lüchun County Yunnan Province of China.

## Conclusion

We constructed the complete mitochondrial DNA sequence of *R. rhodopus* and reconstructed ML and BI phylogenetic trees to explore the taxonomic status and phylogenetic relationships among *R. rhodopus*, *R. bipunctatus* and *R. reinwardtii.* We found the complete mitochondrial sequence of *R. rhodopus* is 15,789 bp in length and consists of 12 protein-coding genes (PCGs), two rRNA, 22 tRNA, and one D-loop. ND5 gene was lacking in the complete mitochondrial sequence of *R. rhodopus.* However, the complex relationship status among *R. rhodopus*, *R. bipunctatus* and *R. reinwardtii* remains unclear. Future studies are needed to sequence more *R. rhodopus* mitochondrial genomes from different geographical regions. Additionally we need to examine *Rhacophorus* specimens from the other genus of Rhacophoridae to further elucidate the phylogenetic structure within the Rhacophoridae.

## Materials and methods

### Sample collection and DNA extraction, sequencing, assembly and annotation

Two frogs were collected from Motuo County (105°4′14.04", 31°49′49.95", 1100 m above sea level [asl]), in the Tibet Autonomous Region of China. Skin samples (a small amount of web between the second and third toes of the hind limbs) were taken after the frogs were anesthetized with MS-222, preserved in 95% ethanol, and stored at − 80 °C. After the skin were taken and the toes of the hind limbs are disinfected with 70% Ethanol, the frogs were released in the same sites. Total mitochondrial genome products were isolated using the Animal Genomic DNA Extraction Kit (TINGKE, Beijing, China). The 350-bp paired-end library construction was applied using the Illumina TruSeqNNanoNDNANLibrary Prep Kit (Illumina, USA), and sequenced with NovaSeq 6000 (Illumina, USA). Approximately 4 Gb of raw data were generated with 150 bp paired-end read lengths. The raw reads were filtered using SOAPnuke^35^, and were assembled using SPAdes^36^(version 3.13.0; parameters: -k 127). Gapcloser (http://soap.genomics.org.cn/, version 1.12) was further employed to fill the gaps. The mitogenome was annotated using MITOS Web Server^37^.

All experimental protocols were approved by the Animal Ethics Committee of Anhui Zoological Society and all experiments followed the recommendations in the ARRIVE guidelines and the American Veterinary Medical Association Guidelines for the Euthanasia of Animals (Ethical proof No. 2022-006).

### Sequence analysis

Each gene was translated into an amino acid sequence using MEGA version 11. The Clustal computer program (version × 1.83) was used to generate the amino acid sequence alignment of each protein coding gene (PCG). Ribosomal RNA (rRNA) genes were identified according to the sequence similarity of a BLAST search, and transfer RNA (tRNA) genes were annotated MITOS^37^. Base composition and codon usage were analyzed using MEGA version 11. The mitochondrial genome sequences were registered in NCBI GenBank with accession numbers (OK165559 and OK181853). For this study, we analyzed the structure of only one sequence (OK181853) due to similarity of the two sequences (Table S1). AT- and GC-skews of the mitochondrial genome were used to detect features of the base composition of nucleotide sequences. The AT-skew and GC-skew were calculated by using the following formulae:

$${\text{AT}} - {\text{skew }} = \, \left( {{\text{A}} - {\text{T}}} \right)/\left( {{\text{A}} + {\text{T}}} \right){\text{ and GC}} - {\text{skew }} = \, \left( {{\text{G}} - {\text{C}}} \right)/\left( {{\text{G}} + {\text{C}}} \right)$$^38^.

### Phylogenetic analysis

To examine the taxonomic status and phylogenetic relationships of these species, we constructed phylogenetic trees based on three types of datasets from GenBank: (1) the complete mitogenome sequences data (21 sequences from 11 species, including 2 outgroup species); (2) the three gene sequences of 12S, 16S and Cytb (396 sequences from 104 species including two *Bufo* species as outgroups); and (3) the two gene sequences of 12S and 16S genes (44 sequences including 27 *R. rhodopus*, 6 *R. bipunctatus*, 7 *R. reinwardtii*, and 4 sequences from 2 *Polypedates* species as outgroups) (Table S5). Firstly, the sequences were aligned and corrected including the use of reverse complement function in BioEdit version 7. Secondly, the sequences were sheared to move out the unaligned bases at both ends, and base composition was counted in MEGA (version 11). Finally the sequences were concatenated using SequenceMatrix software (version 1.8). The optimal nucleotide substitution models were selected with the Akaike Information Criterion (AIC) in jModeltest (version 0.1.1)^39,40^.

Phylogenetic analysis was performed by using maximum-likelihood phylogenies (phyML version 3.0) and Bayesian inference (BI) in MrBayes (version 3.2). BI posterior probabilities were estimated using the Markov Chain Monte Carlo (MCMC) sampling approach. The program initiated with randomly generated trees and ran for 2 × 10^6^ generations in which a total of 2 × 10^4^ trees were sampled at intervals of every 1,000 generations, and then the first 25% of these sampled trees were discarded as burn-in. BI tree was performed using the model GTR + G (nst = mixed; rates = invgamma). An ML tree was constructed using PhyML (version 3.1), and the robustness of the phylogenetic results was tested through bootstrap analysis with 1,000 replicates^41^.

To further explore the evolutionary relationships of *R. rhodopus* in the Rhacophoridae and its regional distribution, the 132 concatenated sequences (12S rRNA + 16S rRNA + Cytb) were used to construct phylogenetic trees (BI and ML) with the optimum model of GTR + I + G (nst = mixed; rates = invgamma). In addition, the concatenated sequences (12S + 16S) were used to construct phylogenetic trees (BI and ML) with the optimum model of TIM2 + I + G (nst = mixed; rates = invgamma). The second and third datasets were analysed using the same strategy as the first dataset.

## Supplementary Information

Supplementary Information 1.

Supplementary Information 2.

Supplementary Information 3.

Supplementary Information 4.

Supplementary Information 5.

## Data Availability

The mitochondrial genome sequences have been deposited in GenBank with accession numbers (OK165559 and OK181853).
