# Supplementary material for: The mitochondrial genome and phylogenetic analysis of Rhacophorus rhodopus
Source: Sci Rep. 2022 Aug 11;12:13693. doi: 10.1038/s41598-022-17814-8 (PMC9372073; doi:10.1038/s41598-022-17814-8)
Supplement: Supplementary file 1 — Supplementary Information 1. [file 41598_2022_17814_MOESM1_ESM.docx]

**Table S1** Characteristics of the mitochondrial genome of *Rhacophorus rhodopus*

|  | **OK181853** | | | **OK165559** | | |  |  |  |  |
| --- | --- | --- | --- | --- | --- | --- | --- | --- | --- | --- |
| **Gene** | **Position** | | **Sizes(bp)** | **Position** | | **Sizes(bp)** | **Codon** | | **Intergenic** | **strand‡** |
|  | **From** | **To** | **Nucleotide** | **From** | **To** | **Nucleotide** | **Start** | **Stop*** | **Nucleotide┼** |  |
| tRNA- Thr | 1 | 71 | 71 | 1 | 71 | 71 |  |  | 15 | H |
| tRNA-Leu (CUN) | 87 | 158 | 72 | 87 | 158 | 72 |  |  | 6 | H |
| tRNA-Pro | 165 | 234 | 70 | 165 | 234 | 70 |  |  | 1 | L |
| tRNA-Phe | 236 | 305 | 70 | 236 | 305 | 70 |  |  | -1 | H |
| 12S rRNA | 305 | 1239 | 935 | 305 | 1239 | 935 |  |  | -1 | H |
| tRNA-Val | 1239 | 1307 | 69 | 1239 | 1307 | 69 |  |  | 3 | H |
| 16S rRNA | 1311 | 2882 | 1572 | 1311 | 2880 | 1570 |  |  | 0 | H |
| tRNA-Leu (UUR) | 2883 | 2954 | 72 | 2881 | 2952 | 72 |  |  | 1 | H |
| ND1 | 2956 | 3922 | 967 | 2954 | 3920 | 967 | ATG | T-- | -6 | H |
| tRNA-Ile | 3917 | 3987 | 71 | 3915 | 3985 | 71 |  |  | -1 | H |
| tRNA-Gln | 3987 | 4057 | 71 | 3985 | 4055 | 71 |  |  | -1 | L |
| tRNA-Met | 4057 | 4125 | 69 | 4055 | 4123 | 69 |  |  | -27 | H |
| ND2 | 4099 | 5140 | 1042 | 4097 | 5138 | 1042 | ATG | T-- | 21 | H |
| tRNA-Trp | 5162 | 5230 | 69 | 5160 | 5228 | 69 |  |  | 0 | H |
| tRNA-Ala | 5231 | 5300 | 70 | 5229 | 5298 | 70 |  |  | 1 | L |
| tRNA-Asn | 5302 | 5374 | 73 | 5300 | 5372 | 73 |  |  | 2 | L |
| OL | 5377 | 5399 | 23 | 5375 | 5397 | 23 |  |  | -1 | L |
| tRNA-Cys | 5399 | 5463 | 65 | 5397 | 5461 | 65 |  |  | 0 | L |
| tRNA-Tyr | 5464 | 5530 | 67 | 5462 | 5528 | 67 |  |  | 4 | L |
| COXI | 5535 | 7088 | 1554 | 5533 | 7086 | 1554 | ATA | AGG | -13 | H |
| tRNA-Ser (UCN) | 7076 | 7146 | 71 | 7074 | 7144 | 71 |  |  | 2 | L |
| tRNA-Asp | 7149 | 7217 | 69 | 7147 | 7215 | 69 |  |  | 0 | H |
| COXII | 7218 | 7911 | 694 | 7216 | 7909 | 694 | ATG | T-- | 0 | H |
| tRNA-Lys | 7912 | 7982 | 71 | 7910 | 7980 | 71 |  |  | 0 | H |
| ATP8 | 7983 | 8147 | 165 | 7981 | 8145 | 165 | ATG | TAA | -10 | H |
| ATP6 | 8138 | 8820 | 683 | 8136 | 8818 | 683 | ATG | TA- | -1 | H |
| COXIII | 8820 | 9604 | 785 | 8818 | 9602 | 785 | ATG | TA- | -1 | H |
| tRNA-Gly | 9604 | 9671 | 68 | 9602 | 9669 | 68 |  |  | 0 | H |
| ND3 | 9672 | 10013 | 342 | 9670 | 10011 | 342 | ATG | TAA | -2 | H |
| tRNA-Arg | 10012 | 10080 | 69 | 10010 | 10078 | 69 |  |  | 0 | H |
| ND4L | 10081 | 10365 | 285 | 10079 | 10363 | 285 | ATG | TAA | -7 | H |
| ND4 | 10359 | 11730 | 1372 | 10357 | 11728 | 1372 | ATG | T-- | -12 | H |
| tRNA-His | 11719 | 11787 | 69 | 11717 | 11785 | 69 |  |  | 0 | H |
| tRNA-Ser (AGY) | 11788 | 11854 | 67 | 11786 | 11852 | 67 |  |  | 4 | H |
| ND6 | 11859 | 12350 | 492 | 11857 | 12348 | 492 | ATG | AGG | 0 | L |
| tRNA-Glu | 12351 | 12418 | 68 | 12349 | 12416 | 68 |  |  | 3 | L |
| Cytb | 12422 | 13559 | 1138 | 12420 | 13557 | 1138 | ATG | T-- | 0 | H |
| D-loop | 13560 | 15789 | 2230 | 13558 | 15742 | 2185 |  |  | 0 | H |
