# Supplementary material for: The mitochondrial genome and phylogenetic analysis of Rhacophorus rhodopus
Source: Sci Rep. 2022 Aug 11;12:13693. doi: 10.1038/s41598-022-17814-8 (PMC9372073; doi:10.1038/s41598-022-17814-8)
Supplement: Supplementary file 2 — Supplementary Information 2. [file 41598_2022_17814_MOESM2_ESM.docx]

**Table S2.** AT/CG skews in the mitochondrial protein-coding genes (PCGs), 2 rRNA genes, D-loop and the complete mitochondrial genome from *Rhacophorus rhodopus*.

| Gene | Size (bp) | A % | T % | G % | C % | (A+T) % | (G+C) % | AT-skew | GC-skew |
| --- | --- | --- | --- | --- | --- | --- | --- | --- | --- |
| ND1 | 967 | 29.89 | 33.51 | 12.41 | 24.20 | 63.39 | 36.61 | -0.057 | -0.322 |
| ND2 | 1042 | 33.01 | 30.52 | 10.75 | 25.72 | 63.53 | 36.47 | 0.039 | -0.411 |
| ND3 | 342 | 24.85 | 35.96 | 15.79 | 23.39 | 60.82 | 39.18 | -0.183 | -0.194 |
| ND4 | 1372 | 28.64 | 32.43 | 12.83 | 26.09 | 61.08 | 38.92 | -0.062 | -0.341 |
| ND4L | 285 | 26.32 | 36.49 | 11.93 | 25.26 | 62.81 | 37.19 | -0.162 | -0.358 |
| ND6 | 492 | 16.26 | 36.59 | 33.94 | 13.01 | 52.95 | 47.05 | -0.385 | 0.446 |
| COX1 | 1554 | 26.71 | 33.53 | 17.57 | 22.20 | 60.23 | 39.77 | -0.113 | -0.117 |
| COX2 | 694 | 30.55 | 30.55 | 15.42 | 23.49 | 61.10 | 38.90 | 0 | -0.207 |
| COX3 | 785 | 27.13 | 32.61 | 16.43 | 23.82 | 59.75 | 40.25 | -0.092 | -0.184 |
| ATP6 | 683 | 28.99 | 34.41 | 9.96 | 26.65 | 63.40 | 36.60 | -0.085 | -0.456 |
| ATP8 | 165 | 32.73 | 35.76 | 7.27 | 24.24 | 68.48 | 31.52 | -0.044 | -0.538 |
| Cytb | 1138 | 26.54 | 27.15 | 13.71 | 32.60 | 53.69 | 46.31 | -0.011 | -0.408 |
| 12S rRNA | 935 | 31.66 | 22.89 | 20.21 | 25.24 | 54.55 | 45.45 | 0.161 | -0.111 |
| 16S rRNA | 1572 | 35.50 | 25.06 | 18.00 | 21.44 | 60.56 | 39.44 | 0.172 | -0.087 |
| D-loop | 2230 | 34.30 | 32.87 | 13.45 | 19.37 | 67.17 | 32.83 | 0.021 | -0.180 |
| 12PCG | 9519 | 27.94 | 32.42 | 14.79 | 24.83 | 60.37 | 39.63 | -0.074 | -0.253 |
| Overall | 15789 | 30.83 | 30.03 | 14.81 | 24.32 | 60.86 | 39.14 | 0.013 | -0.243 |
