# Supplementary material for: The mitochondrial genome and phylogenetic analysis of Rhacophorus rhodopus
Source: Sci Rep. 2022 Aug 11;12:13693. doi: 10.1038/s41598-022-17814-8 (PMC9372073; doi:10.1038/s41598-022-17814-8)
Supplement: Supplementary file 3 — Supplementary Information 3. [file 41598_2022_17814_MOESM3_ESM.docx]

**Table S3.** Codon usage pattern of the 12 mitochondrial protein-coding genes from *Rhacophorus rhodopus*. (The asterisks “*” indicate terminate codon.)

| Codon | Count | RSCU |  | Codon | Count | RSCU |  | Codon | Count | RSCU |  | Codon | Count | RSCU |
| --- | --- | --- | --- | --- | --- | --- | --- | --- | --- | --- | --- | --- | --- | --- |
| **UUU(F)** | **161** | **1.45** |  | UCU(S) | 53 | 1.39 |  | UAU(Y) | 51 | 1.11 |  | UGU(C) | 17 | 1.26 |
| UUC(F) | 61 | 0.55 |  | UCC(S) | 45 | 1.18 |  | UAC(Y) | 41 | 0.89 |  | UGC(C) | 10 | 0.74 |
| **UUA(L)** | **133** | **1.6** |  | UCA(S) | 89 | 2.33 |  | UAA(*) | 3 | 2.4 |  | UGA(W) | 79 | 1.61 |
| UUG(L) | 31 | 0.37 |  | UCG(S) | 5 | 0.13 |  | UAG(*) | 0 | 0 |  | UGG(W) | 19 | 0.39 |
| **CUU(L)** | **100** | **1.2** |  | CCU(P) | 34 | 0.8 |  | CAU(H) | 30 | 0.74 |  | CGU(R) | 9 | 0.56 |
| CUC(L) | 64 | 0.77 |  | CCC(P) | 45 | 1.05 |  | CAC(H) | 51 | 1.26 |  | CGC(R) | 14 | 0.88 |
| **CUA(L)** | **141** | **1.69** |  | CCA(P) | 84 | 1.96 |  | CAA(Q) | 65 | 1.69 |  | CGA(R) | 36 | 2.25 |
| CUG(L) | 31 | 0.37 |  | CCG(P) | 8 | 0.19 |  | CAG(Q) | 12 | 0.31 |  | CGG(R) | 5 | 0.31 |
| **AUU(I)** | **192** | **1.42** |  | ACU(T) | 69 | 1.07 |  | AAU(N) | 56 | 1 |  | AGU(S) | 21 | 0.55 |
| AUC(I) | 79 | 0.58 |  | ACC(T) | 76 | 1.18 |  | AAC(N) | 56 | 1 |  | AGC(S) | 16 | 0.42 |
| **AUA(M)** | **133** | **1.57** |  | **ACA(T)** | **105** | **1.63** |  | AAA(K) | 59 | 1.69 |  | AGA(*) | 0 | 0 |
| AUG(M) | 36 | 0.43 |  | ACG(T) | 7 | 0.11 |  | AAG(K) | 11 | 0.31 |  | AGG(*) | 2 | 1.6 |
| GUU(V) | 47 | 1.26 |  | GCU(A) | 65 | 1.03 |  | GAU(D) | 27 | 0.95 |  | GGU(G) | 37 | 0.79 |
| GUC(V) | 28 | 0.75 |  | **GCC(A)** | **100** | **1.58** |  | GAC(D) | 30 | 1.05 |  | GGC(G) | 45 | 0.96 |
| GUA(V) | 53 | 1.42 |  | GCA(A) | 77 | 1.22 |  | GAA(E) | 54 | 1.4 |  | GGA(G) | 60 | 1.28 |
| GUG(V) | 21 | 0.56 |  | GCG(A) | 11 | 0.17 |  | GAG(E) | 23 | 0.6 |  | GGG(G) | 46 | 0.98 |

Note: *Stop codon.

Bold font indicates preference codon.
