# Supplementary material for: The mitochondrial genome and phylogenetic analysis of Rhacophorus rhodopus
Source: Sci Rep. 2022 Aug 11;12:13693. doi: 10.1038/s41598-022-17814-8 (PMC9372073; doi:10.1038/s41598-022-17814-8)
Supplement: Supplementary file 4 — Supplementary Information 4. [file 41598_2022_17814_MOESM4_ESM.docx]

**Table S4**. Location of features in the D-loop of *Rhacophorus rhodopus.*

| Sequence in D-loop | Start position | Stop position | Length (bp) |
| --- | --- | --- | --- |
| 13.8 tandem repeat units | 119 | 644 | 13×38+32 |
| TAS | 119 | 137 | 19 |
| TAS | 157 | 175 | 19 |
| TAS | 195 | 231 | 19 |
| TAS | 233 | 251 | 19 |
| TAS | 271 | 289 | 19 |
| TAS | 309 | 327 | 19 |
| TAS | 347 | 365 | 19 |
| TAS | 285 | 403 | 19 |
| TAS | 423 | 441 | 19 |
| TAS | 461 | 479 | 19 |
| TAS | 499 | 517 | 19 |
| TAS | 437 | 555 | 19 |
| TAS | 575 | 593 | 19 |
| TAS | 613 | 631 | 19 |
| CSB-1 | 1727 | 1750 | 24 |
| CSB-2 | 1839 | 1857 | 19 |
| CSB-3 | 1882 | 1898 | 17 |
| 12.1 tandem repeat units | 2027 | 2154 | 12×11+4 |
