# Supplementary material for: The mitochondrial genome and phylogenetic analysis of Rhacophorus rhodopus
Source: Sci Rep. 2022 Aug 11;12:13693. doi: 10.1038/s41598-022-17814-8 (PMC9372073; doi:10.1038/s41598-022-17814-8)
Supplement: Supplementary file 5 — Supplementary Information 5. [file 41598_2022_17814_MOESM5_ESM.docx]

**Table S5**. Samples and sequences used in this study (GenBank No).

| Genus | Specific epithet | 12S | 16S | Cyt b | complete mitochondrial |
| --- | --- | --- | --- | --- | --- |
| *Aquixalus* | *odontotarsus* | EF564455 | EF564527 | EU924594 |  |
|  |  |  |  |  |  |
| *Buergeria* | *buergeri* | AB127977 | AB127977 | AB127977 | AB127977 |
|  | *buergeri* | NC008975 | NC008975 | NC008975 | NC008975 |
|  | *japonica* | AB530074 | AB998830 | AB998811 |  |
|  | *japonica* | DQ283055 | DQ283055 | LC133124 |  |
|  | *oxycephala* | EF564443 | EF564514 | EU924592 |  |
|  | *oxycephala* | EU215524 | EU215524 | GQ204548 |  |
|  | *robusta* | AB530075 | AF026370 | JF802981 |  |
|  |  |  |  |  |  |
| *Bufo* | *gargarizans* | KU321581 | KU321581 | KU321581 | KU321581 |
|  | *tibetanus* | JX878885 | JX878885 | JX878885 | JX878885 |
|  |  |  |  |  |  |
| *Chiromantis* | *doriae* | EF564445 | EF564518 | GQ204538 |  |
|  | *nongkhorensis* | AB813158 | KC357609 | GQ204540 |  |
|  | *petersii* | GQ204784 | GQ204733 | GQ204550 |  |
|  | *rufescens* | AF458126 | AF458126 | AY341729 |  |
|  | *rufescens* | AY341622 | KF991282 | GQ204541 |  |
|  | *rufescens* | GQ204775 | KY080106 | KF991323 |  |
|  | *xerampelina* | AB813157 | AB813157 | GQ204551 |  |
|  |  |  |  |  |  |
| *Feihyla* | *palpebralis* | GQ285681 | GQ285681 | EU924597 |  |
|  |  |  |  |  |  |
| *Gracixalus* | *gracilipes* | EF564451 | EF564523 | EU924593 |  |
|  | *jinxiuensis* | EF564452 | KY624584 | EU924599 |  |
|  |  |  |  |  |  |
| *Kurixalus* | *appendiculatus* | AB847125 | AB847125 | KF933134 |  |
|  | *idiootocus* | GQ204744 | GQ204686 | GQ204503 |  |
|  |  |  |  |  |  |
| *Leptomantis* | *gauni* | JN705325 | AF215362 | GQ204531 |  |
|  |  |  |  |  |  |
| *Liuixalus* | *romeri* | EF564464 | KT192638 | EU924598 |  |
|  |  |  |  |  |  |
| *Nyctixalus* | *pictus* | GQ204777 | GQ204726 | GQ204543 |  |
|  | *pictus* | GQ204783 | GQ204732 | GQ204549 |  |
|  |  |  |  |  |  |
| *Philautus* | *abditus* | GQ204760 | KF723226 | GQ204526 |  |
|  | *aurantium* | GQ204756 | KC961078 | GQ204522 |  |
|  | *aurifasciatus* | AY141805 | KY435417 | GQ204520 |  |
|  | *disgregus* | KC961250 | KC961077 | GQ204521 |  |
|  | *ingeri* | JN705354 | JN705385 | GQ204523 |  |
|  | *mjobergi* | JN705349 | KX440533 | GQ204525 |  |
|  | *worcesteri* | GQ204758 | GQ204707 | GQ204524 |  |
|  |  |  |  |  |  |
| *Polypedates* | *braueri* | KT921226 | KT921226 | KT921226 | MK687567 |
|  | *braueri* |  |  |  | NC042797 |
|  | *colletti* | KC961244 | EF624066 | GQ204514 |  |
|  | *cruciger* | GQ204746 | GQ204692 | GQ204508 |  |
|  | *leucomystax* | AB728167 | AB728167 | AB451715 |  |
|  | *leucomystax* | DQ283048 | DQ283048 | JX393485 |  |
|  | *leucomystax* | MW266025 | MW266025 | MK622898 |  |
|  | *macrotis* | KU840482 | KU840554 | GQ204512 |  |
|  | *megacephalus* | AY458598 | AY458598 | AY458598 | AY458598 |
|  | *megacephalus* | MH936677 | MH936677 | MH936677 | MH936677 |
|  | *megacephalus* | NC043955 | NC043955 | NC043955 | NC043955 |
|  | *mutus* | AY880608 | KR828028 | MK622900 |  |
|  | *otilophus* | AB907717 | AB907717 | GQ204513 |  |
|  |  |  |  |  |  |
| *Pseudophilautus* | *alto* | GQ204738 | GQ204677 | GQ204494 |  |
|  | *amboli* | JX092728 | KU169982 | JX092788 |  |
|  | *cavirostris* | FJ788137 | FJ788156 | GQ204493 |  |
|  | *kani* | JX092754 | KP939071 | JX092820 |  |
|  | *lunatus* | FJ788150 | FJ788169 | GQ20449 |  |
|  | *microtympanum* | DQ019587 | DQ019604 | GQ204495 |  |
|  | *mittermeieri* | GQ204741 | GQ204681 | GQ204498 |  |
|  | *poppiae* | FJ788136 | GQ204670 | GQ204487 |  |
|  | *schmarda* | AY880617 | AY880530 | GQ204486 |  |
|  | *simba* | GQ204740 | GQ204679 | GQ204496 |  |
|  | *stuarti* | FJ788139 | GQ204672 | GQ204489 |  |
|  | *zorro* | FJ788147 | FJ788147 | GQ204488 |  |
|  |  |  |  |  |  |
| *Raorchestes* | *akroparallagi* | JX092726 | JX092650 | JX092786 |  |
|  | *anili* | JX092729 | JX092700 | JX092790 |  |
|  | *beddomii* | JX092731 | JX092653 | JX092793 |  |
|  | *bobingeri* | *JX092733* | KM596529 | JX092795 |  |
|  | *charius* | AF249032 | AF249062 | AF249095 |  |
|  | *charius* | JX092735 | JX092707 | GQ204500 |  |
|  | *charius* | JX092736 | KM596548 | JX092798 |  |
|  | *charius* | KU170010 | KU169985 | JX092799 |  |
|  | *chotta* | JX092737 | JX092656 | JX092800 |  |
|  | *chromasynchysi* | JX092738 | JX092667 | JX092802 |  |
|  | *coonoorensis* | JX092740 | JX092716 | JX092805 |  |
|  | *crustai* | JX092742 | JX092677 | JX092806 |  |
|  | *glandulosus* | JX092744 | JX092665 | JX092810 |  |
|  | *graminirupes* | JX092772 | JX092649 | JX092812 |  |
|  | *griet* | AY706108 | KU169990 | JX092814 |  |
|  | *jayarami* | JX092750 | JX092686 | JX092816 |  |
|  | *johnceei* | JX092751 | JX092679 | JX092817 |  |
|  | *kaikatti* | JX092752 | JX092718 | JX092822 |  |
|  | *luteolus* | JX092756 | JX092659 | JX092823 |  |
|  | *marki* | JX092757 | JX092719 | JX092825 |  |
|  | *nerostagona* | JX092760 | JX092661 | JX092830 |  |
|  | *ochlandrae* | JX092743 | JX092666 | JX092831 |  |
|  | *ponmudi* | JX092762 | JX092651 | JX092832 |  |
|  | *signatus* | AY141795 | AY141841 | AY708169 |  |
|  | *signatus* | JX092764 | KM596562 | GQ204501 |  |
|  | *sushili* | JX092766 | JX092684 | JX092844 |  |
|  | *theuerkaufi* | JX092767 | JX092693 | JX092845 |  |
|  | *tinniens* | AY706112 | JX092715 | JX092846 |  |
|  | *travancoricus* | JX092776 | JX092721 | JX092847 |  |
|  |  |  |  |  |  |
| *Rhacophorus* | *annamensis* | AF458143 | LC548739 | GQ204534 |  |
|  | *bipunctatus* | AF458144 | AF458144 |  |  |
|  | *bipunctatus* | AY843750 | AY843750 | EU924602 |  |
|  | *bipunctatus* | GQ204767 | GQ204716 | GQ204533 |  |
|  | *bipunctatus* | GQ204767 | GQ204767 |  |  |
|  | *bipunctatus* | JX219444 | JX219444 |  |  |
|  | *bipunctatus* | JX219445 | JX219445 |  |  |
|  | *calcaneus* | GQ204770 | KX139181 | GQ204536 |  |
|  | *catamitus* | MF066241 | MF066241 | MF066241 |  |
|  | *malabaricus* | GU136096 | AF249050 | AF249094 |  |
|  | *modestus* | MF066238 | MF066238 | MF066238 |  |
|  | *pardalis* | KU840489 | MF004470 | GQ204528 |  |
|  | *poecilonotus* | MF066240 | MF066240 | MF066240 |  |
|  | *reinwardtii* | EF564498 | EF564570 | EU924615 |  |
|  | *reinwardtii* | EF564499 | EF564571 | GQ204530 |  |
|  | *reinwardtii* | GQ204771 | GQ204720 |  |  |
|  | *reinwardtii* | JX219443 | JX219443 |  |  |
|  | *rhodopus* | EF564500 | EF564572 |  |  |
|  | *rhodopus* | EF564501 | EF564573 |  |  |
|  | *rhodopus* | EF564502 | JX219440 |  |  |
|  | *rhodopus* | EF564503 | EF564575 |  |  |
|  | *rhodopus* | EF564504 | EF564576 |  |  |
|  | *rhodopus* | EF564505 | EF564577 |  |  |
|  | *rhodopus* | EF564506 | EF564578 |  |  |
|  | *rhodopus* | EF564507 | EF564579 |  |  |
|  | *rhodopus* | EF564508 | EF564580 |  |  |
|  | *rhodopus* | EU215529 | EU215529 |  |  |
|  | *rhodopus* | EU215531 | EU215531 |  |  |
|  | *rhodopus* | JX219439 | JX219439 |  |  |
|  | *rhodopus* | JX219440 | EF564574 |  |  |
|  | *rhodopus* | JX219441 | JX219441 |  |  |
|  | *rhodopus* | JX219442 | JX219442 | EU924616 |  |
|  | *rhodopus* | LC386573 | LC386573 |  |  |
|  | *rhodopus* | OK165559 | OK165559 | OK165559 | **OK165559** |
|  | *rhodopus* | OK181853 | OK181853 | OK181853 | **OK181853** |
|  |  |  |  |  |  |
| *Rohanixalus* | *vittatus* | AF458131 | AF458131 | EU924596 |  |
|  | *vittatus* | DQ283134 | DQ283134 | GQ204539 |  |
|  |  |  |  |  |  |
| *Taruga* | *eques* | AY880489 | GQ204689 | GQ204505 |  |
|  | *fastigo* | AY141802 | GQ204690 | GQ204506 |  |
|  | *longinasus* | GQ204745 | GQ204691 | GQ204507 |  |
|  |  |  |  |  |  |
| *Theloderma* | *asperum* | LC012856 | LC012856 | EU924618 |  |
|  | *asperum* | GQ204776 | KR828081 | GQ204542 |  |
|  | *bicolor* | KJ802915 | KT461923 | KF991324 |  |
|  | *rhododiscus* | KY495631 | DQ283393 | EU924619 |  |
|  |  |  |  |  |  |
| *Zhangixalus* | *arboreus* | LC565708 | LC565708 | LC565708 | LC565708 |
|  | *burmanus* | EF564496 | EF564568 | EU924617 |  |
|  | *chenfui* | EF564465 | EF564537 | EU924603 |  |
|  | *chenfui* | JX219432 | JX219432 | GQ204529 |  |
|  | *dennysi* | KM035412 | KM035412 | KM035412 | KM035412 |
|  | *dennysi* | KT191129 | KT191129 | KT191129 | KT191129 |
|  | *dennysi* | KX233869 | KX233869 | KX233869 | KX233869 |
|  | *dennysi* | NC027452 | NC027452 | NC027452 | NC027452 |
|  | *dugritei* | KU840491 | KU840565 | EU924605 | MZ712011 |
|  | *dugritei* | MZ712011 | MZ712011 | MZ712011 |  |
|  | *dulitensis* | JX219434 | JX219434 | GQ204532 |  |
|  | *feae* | EF564474 | EF564546 | EU924606 |  |
|  | *hui* | EU924627 | EU924622 | EU924607 |  |
|  | *maximus* | EF564476 | EF564548 | EU924608 |  |
|  | *minimus* | EF564489 | EF564560 | EU924609 |  |
|  | *nigropunctatus* | EF564491 | EF564563 | EU924611 |  |
|  | *omeimontis* | EU215535 | EU215535 | EU924612 |  |
|  | *omeimontis* | MN427892 | MN427892 | MN427892 | MN427892 |
|  | *omeimontis* | NC046387 | NC046387 | NC046387 | NC046387 |
|  | *pingbianensis* | EF564495 | EF564567 | EU924613 |  |
|  | *pingbianensis* | EU924629 | EU924624 | EU924614 |  |
|  | *schlegelii* | AB202078 | AB202078 | AB202078 | AB202078  NC007178 |
|  | *schlegelii* | NC007178 | NC007178 | NC007178 |  |
